# Polymer Chemistry in Living Cells

**DOI:** 10.1021/acs.accounts.2c00420

**Published:** 2022-09-30

**Authors:** Zhixuan Zhou, Konrad Maxeiner, David Y. W. Ng, Tanja Weil

**Affiliations:** Max Planck Institute for Polymer Research, Ackermannweg 10, 55128 Mainz, Germany

## Abstract

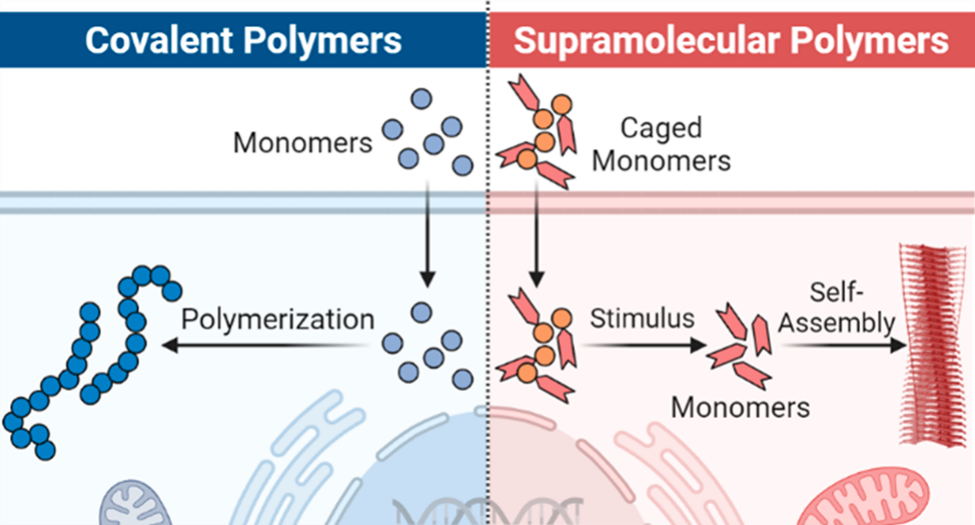

The polymerization of biomolecules is a central operation in biology that connects molecular signals with proliferative and information-rich events in cells. As molecules arrange precisely across 3-D space, they create new functional capabilities such as catalysis and transport highways and exhibit new phase separation phenomena that fuel nonequilibrium dynamics in cells. Hence, the observed polymer chemistry manifests itself as a molecular basis leading to cellular phenotypes, expressed as a multitude of hierarchical structures found in cell biology. Although many milestone discoveries had accompanied the rise of the synthetic polymer era, fundamental studies were realized within a closed, pristine environment and that their behavior in a complex multicomponent system remains challenging and thus unexplored. From this perspective, there is a rich trove of undiscovered knowledge that awaits the polymer science community that can revolutionize understanding in the interactive nanoscale world of the living cell.

In this Account, we discuss the strategies that have enabled synthetic polymer chemistry to be conducted within the cells (membrane inclusive) and to establish monomer design principles that offer spatiotemporal control of the polymerization. As reaction considerations such as monomer concentration, polymer growth dynamics, and reactivities are intertwined with the subcellular environment and transport processes, we first provide a chemical narrative of each major cellular compartment. The conditions within each compartment will therefore set the boundaries on the type of polymer chemistry that can be conducted. Both covalent and supramolecular polymerization concepts are explored separately in the context of scaffold design, polymerization mechanism, and activation. To facilitate transport into a localized subcellular space, we show that monomers can be reversibly modified by targeting groups or stimulus-responsive motifs that react within the specific compartment. Upon polymerization, we discuss the characterization of the resultant polymeric structures and how these phase-separated structures would impact biological processes such as cell cycle, metabolism, and apoptosis. As we begin to integrate cellular biochemistry with in situ polymer science, we identify landmark challenges and technological hurdles that, when overcome, would lead to invaluable discoveries in macromolecular therapeutics and biology.

## Key References

- Zhou, Z.; Maxeiner, K.; Moscariello, P.; Xiang, S.; Wu, Y.; Ren, Y.; Whitfield, C. J.; Xu, L.; Kaltbeitzel, A.; Han, S.; Mucke, D.; Qi, H.; Wagner, M.; Kaiser, U.; Landfester, K.; Lieberwirth, I.; Ng, D. Y. W.; Weil, T. In Situ Assembly of Platinum(II)–Metallopeptide Nanostructures Disrupts Energy Homeostasis and Cellular Metabolism. *J. Am. Chem. Soc.***2022**, *144*, 12219–12228.^1^*Cooperative intermolecular forces promoted by square planar Pt(II) complex-conjugated β-amyloid peptides were shown to form intracellular nanofibers. The presence of these near-infrared emissive nanofibers disrupts cellular metabolism and impacts ATP-dependent pathways efficiently*.
- Pieszka, M.; Han, S.; Volkmann, C.; Graf, R.; Lieberwirth, I.; Landfester, K.; Ng, D. Y. W.; Weil, T. Controlled Supramolecular Assembly Inside Living Cells by Sequential Multistaged Chemical Reactions. *J. Am. Chem. Soc.***2020**, *142*, 15780–15789.^2^*This article describes the use of an oxidatively coupled O,N-rearrangement reaction to program how intermolecular forces propagate into hierarchical structures within the complex cellular environment*.
- Ng, D. Y. W.; Vill, R.; Wu, Y.; Koynov, K.; Tokura, Y.; Liu, W.; Sihler, S.; Kreyes, A.; Ritz, S.; Barth, H.; Ziener, U.; Weil, T. Directing intracellular supramolecular assembly with N-heteroaromatic quaterthiophene analogues. *Nat. Commun.***2017**, *8*, 1850.^3^*The large spectral shift of substituted oligothiophenes upon self-assembly was exploited to grant real-time visualization of supramolecular events in cells. Correlation with subcellular localization was found to be dependent on the N-heteroaromatic substitution*.
- Wang, T.; Wu, Y.; Kuan, S. L.; Dumele, O.; Lamla, M.; Ng, D. Y. W.; Arzt, M.; Thomas, J.; Mueller, J. O.; Barner-Kowollik, C.; Weil, T. A Disulfide Intercalator Toolbox for the Site-Directed Modification of Polypeptides. *Chem. Eur. J.***2015**, *21*, 228–238.^4^*Using self-reporting tetrazole-maleimide photoclick chemistry, ultrafast polymer conjugation reactions in the cytosol can be performed endogenously and tracked via fluorescence*.

## Introduction

The precise control of molecular interactions in complex environments represents a key characteristic of living systems. Nature’s grand scheme for the molecular evolution of life could be observed across structural hierarchies, from atomic level recognition in ion channels, to catalysis at diffusional rates, to the formation of superstructures programmed by protein assemblies. Particularly at the nanoscale, molecular components are created on demand and behave dynamically to meet a precise requirement of the system at any given time.^5^ This intrinsic machinery has led to significant efforts to analyze how an interactive molecular system becomes living and builds synthetic macromolecular architectures directly in cells. The former question is invested extensively in the exploration of the origin of life, where synthetic cellular prototypes are built to allow a systematic input of molecular and nanoscale components until primitive life-like behavior can be demonstrated. However, the latter aspect of modulating biology by in situ polymer chemistry is often hampered by the lack of chemical methods to control and to overcome the dynamic and crowded intracellular environment.

Unlike the closed, static system where most synthetic chemistry is performed in, the processes within living cells dictate how reactive molecules are distributed and the dynamics of local reaction media. Therefore, the concentration of an artificially introduced molecule at any given time is heterogeneous and subject to a variety of biological factors such as the rate of internalization/extrusion, the mode of transport (active/passive), and phases of cell growth. The next important understanding is the division of biological environments into compartments and organelles, where molecules typically encounter multiple interfacial forces during transport. Once intracellular transport is successful, compartmentalization provides an exploitative basis for molecular enrichment and isolation, offering new possibilities that are ideal for building nanoscale structures. With this knowledge, synthetic alterations to cellular behavior will no longer be limited to small-molecule chemistry and that the field of polymer chemistry will soon be able to bring forth new fundamental and application-driven concepts in biological science.

## Chemical Perspective of the Cell

**Figure 1:**
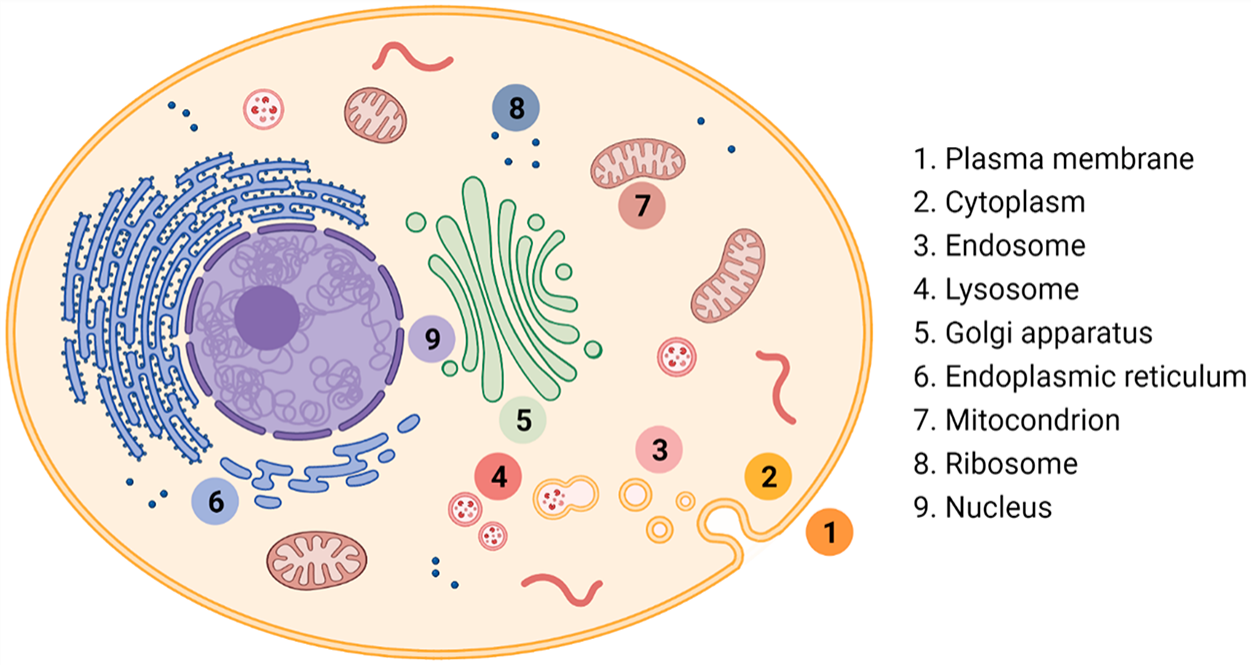
Key cellular compartments.

Each subcellular compartment can be viewed as a reaction vessel with a set of generic predefined conditions in cell biology (Figure 1). The aqueous medium and the presence of biomolecular components are ubiquitous, which means that designated chemical reactions must be selective toward functional groups such as alcohols, thiols, amines, carboxylic acids, and metal ions. Although a significant amount of inspiration can be derived from small-molecule biorthogonal chemistry,^6^ the modification of cell organelles through polymerization techniques or the construction of ordered architectures in cells requires several additional considerations. A key challenge is to keep macromolecular-sized reactive partners or polymerization monomers chemically active in the crowded, heterogeneous environment of the cell and control the monomer concentration required for polymerization.

The cell plasma membrane is the first organelle and barrier encountered by any synthetic molecule that must be traversed to gain access to the intracellular system. The membrane features analogues of phospholipids, glycolipids, and sterols assembled into a continuous bilayer structure punctuated by transmembrane proteins that serve both signaling and transport functions.^7^ Functionalization strategies include lipophilic anchor groups to prepare the membrane for subsequent reactions or target specific transmembrane proteins via ligand–receptor interactions.^8−10^ Depending on the exact location, it is important to recognize that reactive moieties on the plasma membrane remain stable only between 1 and 4 h as parts of the membrane partake in vesicular uptake processes and recycling.^8^ Design principles that target the cell membrane can also be applied, to varying degrees, to intracellular membrane-bound organelles such as the mitochondria, the endoplasmic reticulum (ER), the Golgi apparatus, and the nucleus.

Targeting reactive molecules within the cell to a specific organelle usually requires an additional handle that has a chemical or biological affinity for it. These handles range from small entities like the triphenylphosphonium cation (TPP, mitochondria),^11^*p*-toluolsulfonamides (ER/Golgi),^12^ to targeting peptides like the nuclear localization sequence (NLS).^13^ Thus, once the designated molecule reaches the target organelle, the local environment dictates the reaction conditions. Elevated glutathione (GSH) concentration in the cytosol of cancer cells forms a reductive environment, reactive oxygen species (ROS) near the mitochondria could be used for oxidation reactions, and the acidity of endosomes (pH 6.0–6.5) and lysosomes (pH 5.5) could be used for controlled bond cleavage reactions; some of these conditions are also frequently exploited in drug delivery.^14,15^ Advanced considerations can involve using specific enzymes at local, subcellular conditions (i.e., cathepsins for lysosomes, glucose-6-phosphatase for ER) to initiate a chemical reaction.^15−17^ Therefore, the dynamics and lifetime of different cellular environments need to be considered when selecting a polymerization technique that allows polymer formation at distinct locations within cells to achieve the desired biological outcome.

## Conjugation of Synthetic Polymer to the Cell Surface

Natural covalent polymers are ubiquitous structural components forming the living system, and they are responsible for carrying out many biological functions. The capabilities to conduct polymerization reactions of synthetic macromolecules in the living system offer unique synthetic approaches to understand and modulate biological processes, to form new cellular compartments, and to confer novel functionalities to the living cell.

**Figure 2:**
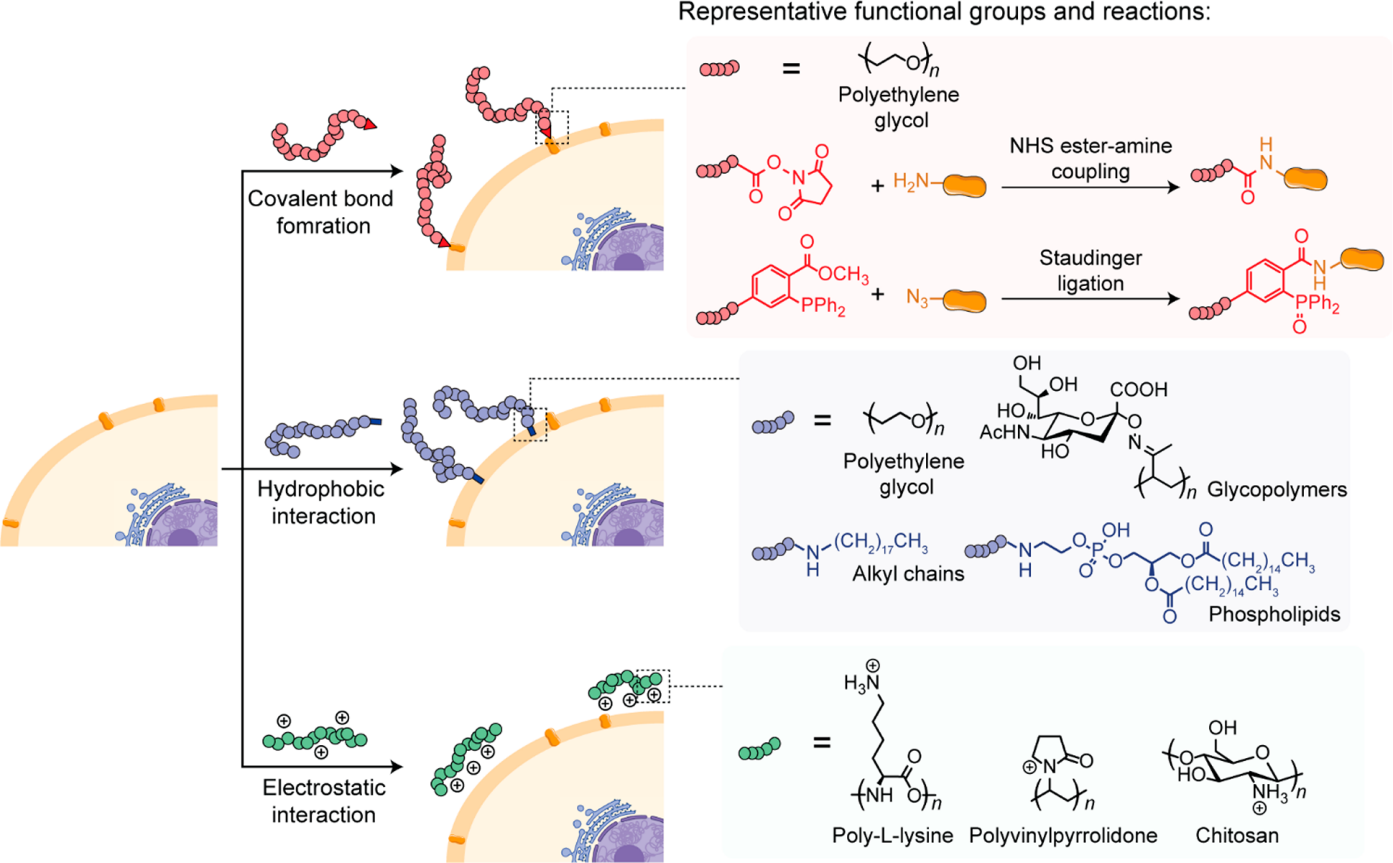
Schematic illustration of synthetic polymer conjugation to the cell surface.

In most eukaryotes and some prokaryotes, the cell surfaces consist of proteins and polysaccharides present on the exterior of the cell membrane to provide support and protection. One of the first examples was the straightforward coupling of a preformed synthetic polymer to the cell surface components. This process relies on the interaction between the synthetic polymer and the cell surface components with high efficiency under physiological conditions (Figure 2).

The proteins on the cell surface are major targets for attaching already formed polymer chains. The synthetic polymers are often activated with *N*-hydroxyl-succinimidyl ester (NHS) or cyanuric chloride groups for covalent bond formation with the lysine or cysteine residue on the proteins.^18,19^ Additional functional groups such as the azido group and HaloTag protein could also be introduced to the cell surface proteins through protein engineering and subsequently utilized for conjugating synthetic polymers only to the protein of interest in a biorthogonal fashion.^20−23^ This approach provides better specificity and modularity compared to targeting of widely abundant natural amino acid residues on all proteins on the cell surface. Although covalent conjugation offers excellent stability toward degradation, there is a potential for disrupting the cellular physiology and could interfere with important cellular functions governed by the cell surface constituents.^22^

The lipid bilayer of the cell membrane is the primary target for the noncovalent integration of synthetic polymers via hydrophobic interactions.^8−10^ This approach has been demonstrated using PEG and glycopolymers. Beyond hydrophobic interactions, synthetic polymers can be deposited onto the negatively charged cell surface through electrostatic adsorption. Direct deposition of polycationic polymers, including poly-l-lysines, polyvinylpyrrolidones, and glycopolymers, can often be detrimental to cell viability.^24^ Hence, the cationic polymers are often copolymerized with biocompatible PEG moieties to mediate their interactions with the cell surface and thus reduce cytotoxicity.^25^ The deposition of cationic polymers on the cellular surface can enable further chemistry utilizing electrostatic interactions.^24−27^

## In Situ Polymer Synthesis on the Cell Surface

Polymer synthesis at the cell surface faces significant limitations because conventional polymerization reactions are often cytotoxic, including the use of reactants and conditions that are harmful to cells and the generation of reactive species with cross-reactivity to cell surface components. Therefore, initial polymerization methods rely on precursors that are known to be benign in the cellular environment.

Polydopamine (PD) is a bioinspired and biocompatible polymer that resembles certain features of mussel foot proteins and is known for its rapid self-polymerization and its adhesive properties.^28^ Immersing live yeast cells in a dopamine solution leads to the formation of a PD shell on the cell surface as a result of covalent bond formation (Schiff-base and Michael-type reactions) between PD and amine/thiol groups of the cell wall proteins.^28^ Recently, yeast cells coated with PD-based atom transfer radical polymerization (ATRP) initiators were obtained by priming the cell with a solution of dopamine–initiator conjugate.^29^ The coated cells were then placed in an aqueous solution containing the catalyst, ligand, reducing agent, and sodium methacrylate monomer under ambient conditions to initiate the polymerization reaction.

**Figure 3:**
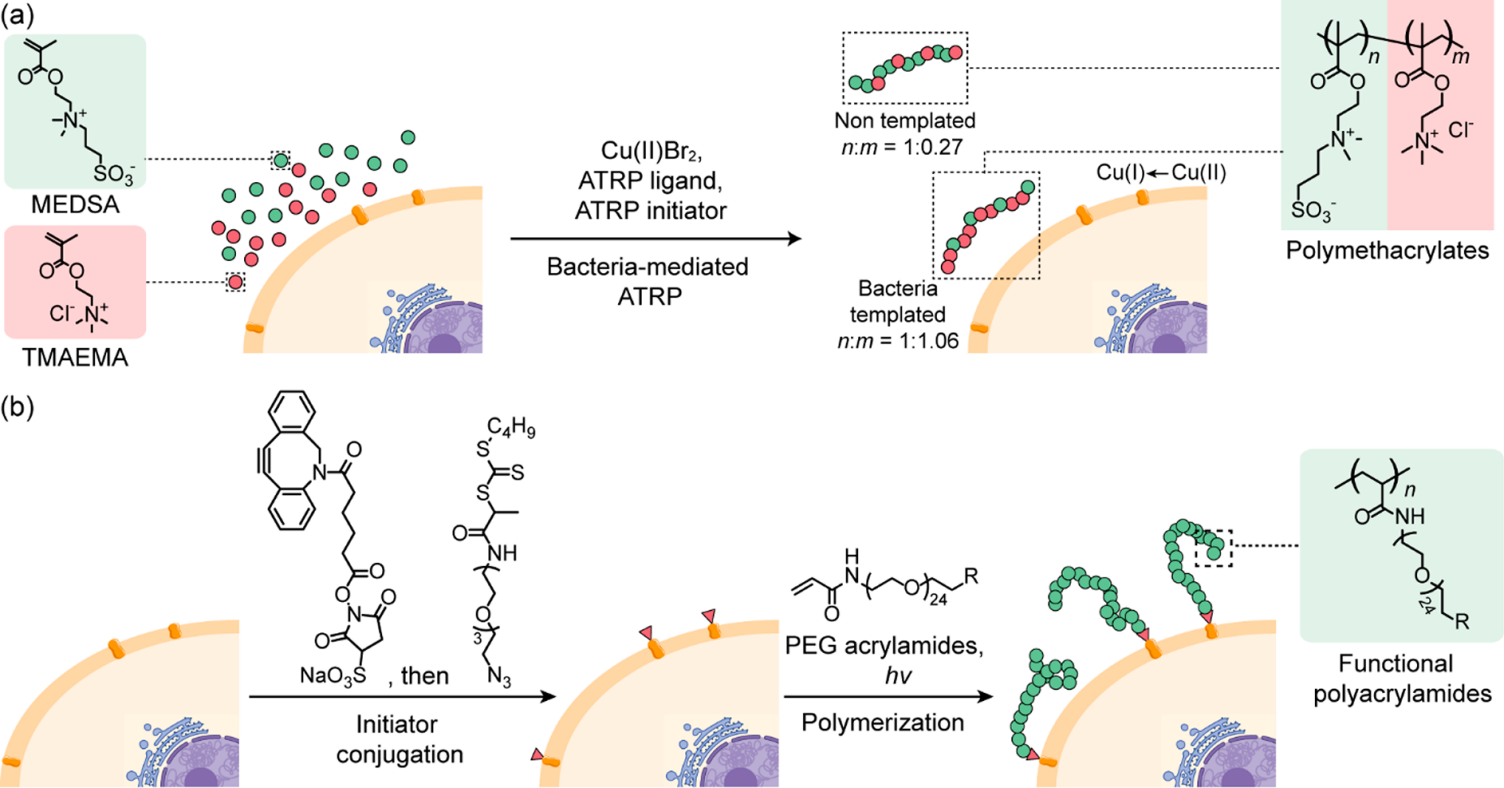
Representative reactions for in situ polymer synthesis on the cell surface. (a) Bacteria-templated ATRP.^30^ (b) PET-RAFT initiated by initiators covalently attached to the yeast cell surface.^34^

Although living cells are generally considered a hurdle for the polymerization reactions, specific cellular functions can be harnessed to synthesize polymers possessing emergent properties not accessible through conventional reaction conditions.^30−33^ For example, the Cu(I) species generated by the copper homeostasis in *E. coli* can be exploited as a catalyst for the synthesis of acrylic polymers by ATRP (Figure 3a).^30^*E. coli* was incubated with cationic 2-(methacryloyloxy)-*N*,*N*,*N*-trimethylethanaminium chloride (TMAEMA) and zwitterionic sulfobetaine, 2-(*N*-3-sulfopropyl-*N*,*N*-dimethylammonium) ethyl methacrylate (MEDSA), monomers before addition of Cu(II)Br_2_, ATRP ligands, and ATRP-initiator to the cell suspension. The polymerization was initiated by Cu(I) species generated in situ by the reducing environment on the bacterial surfaces. The polymers grew directly on the bacterial surface exhibited specific and robust binding to the templating bacteria. Similarly, the iron-reducing systems of bacteria have been used to trigger iron-mediated ATRP of a variety of hydrophilic polymers with well-defined molecular weights on the bacterial surface.^33^

Polymerization reactions can also be initiated from a synthetic molecule preinserted on the cell surface. This approach can provide better control over polymer properties, including monomer types, polymer length, and functionalities. Photoinduced electron transfer-reversible addition–fragmentation chain-transfer polymerization (PET-RAFT) was successfully performed on cell surfaces using this strategy (Figure 3b).^34^ Yeast cells were decorated via an amidation reaction between NHS-activated dibenzocyclooctyl and the free amino groups on the cell surface, where the azide-functionalized initiator was subsequently introduced by azide–alkyne cycloaddition. The polymerization was then performed under a mild blue light source using Eosin Y/triethanolamine as the photocatalytic system and methoxy-PEG acrylamide as the monomer. A variety of functional groups can be incorporated using functionalized monomers, and the developed polymerization strategy allows for a higher grafting density compared to coupling preformed polymers to the cell surface. The polymerization can also be performed on mammalian cells by noncovalently anchoring the initiators to the cell membrane through a noncharged DPPE-mimicking tail.

These studies on cell surface polymerization reactions have focused on gaining a fundamental understanding of the cell surface properties and to regulate cell-based interactions.^18−22^ Polymers grown on the cell surface can be considered as synthetic mimics of the extracellular matrix, which enables diverse features, such as facilitating the electron transfer processes, cell-specific binding, and intercell communication.^30−32^ With the emergence of more efficient, less cytotoxic, and mild polymerization strategies, diverse biomedical applications have been realized, including cell-based therapy, drug delivery, tissue engineering, and immune modulation.^26,27^

## Intracellular Polymerization and Polymer Conjugation

The complex chemical environment and biological processes in living cells present both challenges and opportunities to chemists. Current research has shown that polymerization reactions can be conducted in living cells with excellent versatility in terms of polymer composition, reaction condition, and chemical functionalities. However, the intracellular formation of synthetic polymers faces several challenges due to the presence of a large variety of molecules that can perturb or quench the intracellular reactions. In addition, complex intracellular processes such as cellular uptake and subcellular distribution of the reactants must be considered when planning chemical reactions within cells.

**Figure 4:**
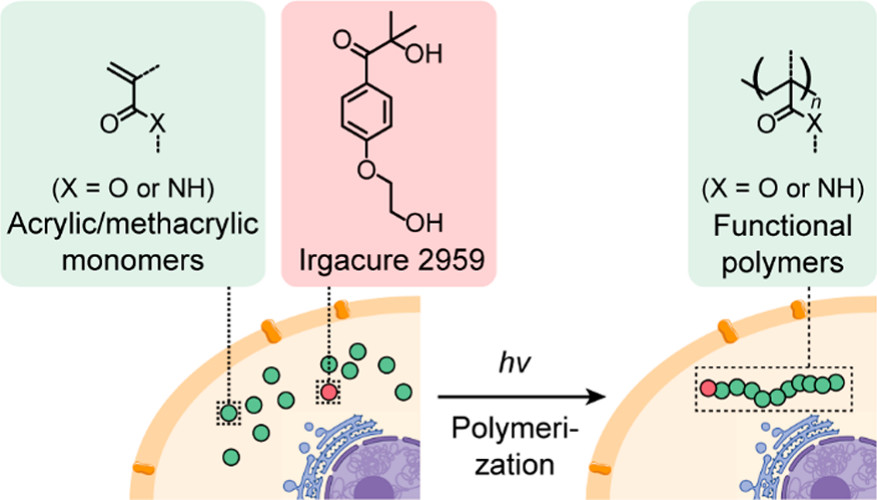
Illustration of a light-mediated intracellular free radical polymerization reaction.^35^

Light provides energy to initiate photoreactions without affecting most biomolecules present within cells. Moreover, it offers spatiotemporal control, which is particularly useful for directing reactions in real time at distinct locations within the living cell. For example, light-initiated tetrazole–ene cycloaddition of a membrane-penetrating methoxypolyethylene glycol maleimide and a peptide carrying a tetrazole group has been demonstrated by our group.^4^ The peptide hormone somatostatin (SST) modified with a tetrazole group was internalized via endocytosis into cells carrying SST receptors, and bioconjugation with methoxypolyethylene glycol maleimide proceeded without compromising cell viability. Recently, free radical photopolymerization approach was successfully accomplished in cells (Figure 4).^35^*N*-(2-Hydroxypropyl) methacrylamide (HMPA) was selected as a model compound because of its cell compatibility. Photopolymerization was initiated in eukaryotic cells incubated with HMPA and the photoinitiator (Irgacure 2959). This approach was then extended to biocompatible acrylic and methacrylic monomers. The cells containing the intracellular generated polymers exhibited a larger residual gap area in wounds compared to untreated cells in a wound-healing assay, suggesting reduced cell motility upon polymerization.

Although covalent polymerization and conjugation inside cells is often challenged by concentration gradients and transport pathways, it offers unique approaches to materials that combine the efficient biodistribution of small molecules and the prolonged retention of macromolecules inside cells. Moreover, polymers can elicit bioactivity at a nanostructural level, creating a new modality to intervene cell cycles, stress responses, and differentiation. Hence, the chemical and biochemical insight obtained through cellular behavior enables a better understanding of the biological effects of the synthetic polymers and promotes functions that are beneficial for diverse applications, including imaging, cancer therapy, and modulation of behavior in living animals.^36−40^

## Supramolecular Polymerization within the Cells (Membrane Inclusive)

**Figure 5:**
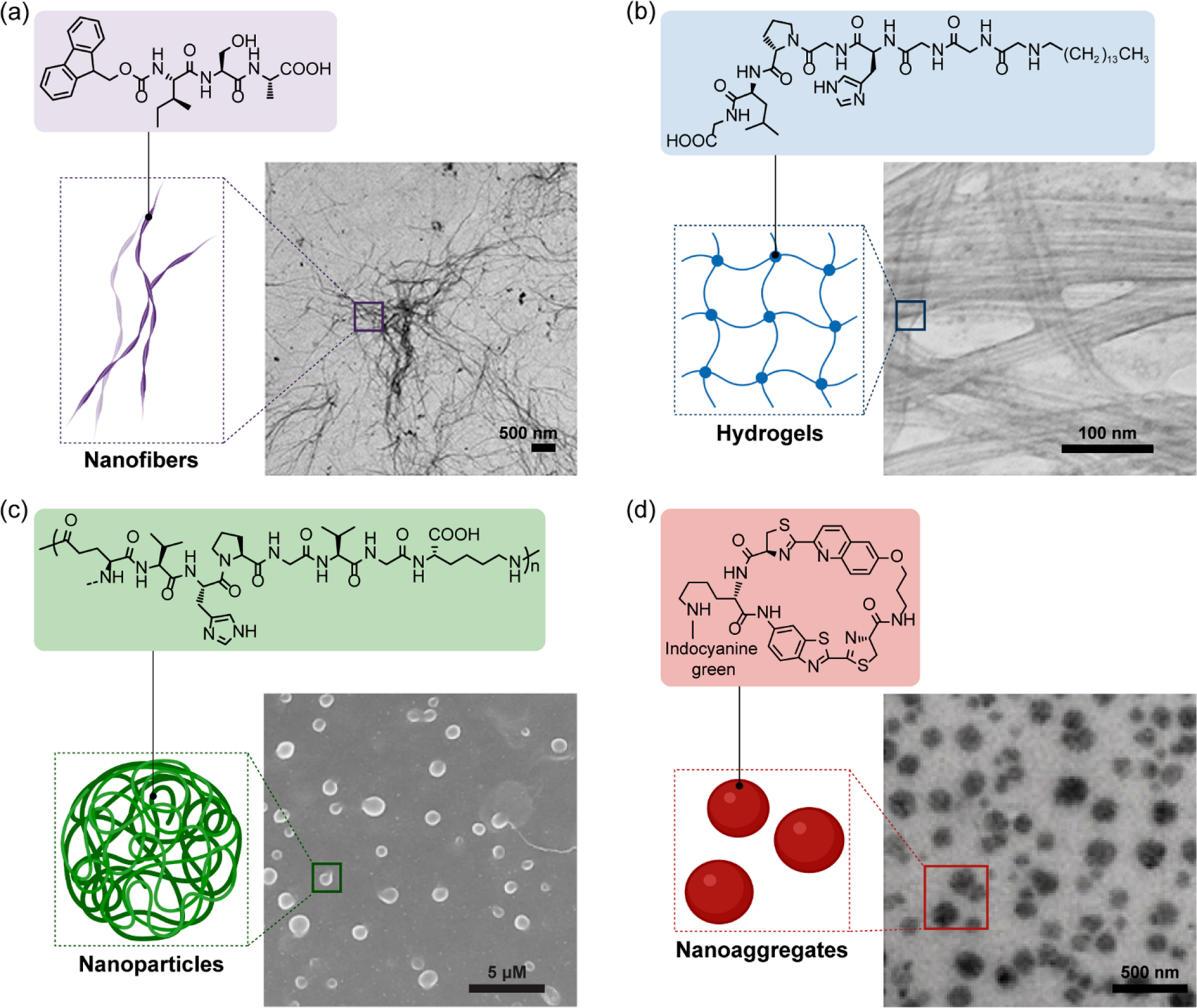
Various morphologies of nanostructures obtainable by controlled assembly within cells. (a) Nanofibers. Adapted with permission from ref (1). Copyright 2020 American Chemical Society (b) Hydrogels. Adapted with permission from ref (43). Copyright 2015 American Chemical Society. (c) Nanoparticles. Adapted with permission from ref (44). Copyright 2017 Springer Nature. (d) Nanoaggregates. Adapted with permission from ref (45). Copyright 2019 John Wiley and Sons.

In biology, supramolecular interactions play an essential role in the dynamics of functional architectures on every length scale, from the base pairing of DNA to the formation of complex nanostructures such as microtubuli or actin filaments to macroscopic fibers like silk.^41^ Regardless of the differences in scale, the biological system is optimized and compartmentalized in a way to allow mass transport of molecules and reaction efficiencies far beyond the reach of analogous synthetic counterparts.^5^ This feature works primarily by programming noncovalent interactions such as hydrogen bonds, van der Waals, and π–π forces over precise 3-D space to form a unique interface that only recognizes a designated partner that possesses the correct conformational and energy requirements.^42^ In contrast to covalent polymerization, supramolecular polymerization can be highly directional and reversible, leading to a greater variety of properties affecting the binding dynamics (e.g., stimulus responsiveness, equilibrium-driven lifetimes, biodegradability). Therefore, critical concentrations of supramolecular polymerization can often be easily tailored to a low micromolar regime where covalent polymerization would be difficult to achieve, especially in a complex environment. As a result, by tailoring the assembly mechanisms to physiological conditions, a variety of morphologies including nanoparticles/aggregates, fibers, and gel networks were generated in situ (Figure 5, Table 1).^2,43−45^

**Table 1:** Overview for Supramolecular Polymerization Strategies within the Cellular Space

monomer	activation process/stimulus	location of self-assembly	nanostructure	application	ref
aromatic macrocycle	multistep	cytoplasm	nanoparticles	imaging of enzymatic activity	(47,57,66)
	(1) enzymatic cleavage		nanoaggregates		
	(2) pH				
	(3) reductive environment (GSH)				
peptide (FF motif)	enzymatic dephosphorylation	endoplasmic reticulum	nanofibers	inducing apoptosis of cancer cells	(62)
		mitochondria	nanoaggregates		
peptide	(1) enzyme-catalyzed polymerization	cytoplasm	nanoparticles	inducing apoptosis of cancer cells	(44)
	(2) temperature		random coil		
			hydrogel		
polymer–peptide conjugate	enzymatic cleavage of peptide	cytoplasm	nanoaggregates	imaging of enzymatic activity	(67)
poly(ethylene glycol) diacrylate monomer	light-induced polymerization	cytoplasm	hydrogel	cell fixation	(61)
oligothiophenes	temperature	cytoplasm	nanoaggregates	intracellular imaging	(3)
		mitochondria			
oligothiophenes		cytoplasm	fluorescent collagen fibrils	intracellular imaging	(50)
isopeptide	multistep intramolecular rearrangement	cytoplasm	nanofibers	disrupting cellular metabolism and inducing apoptosis of cancer cells	(1,2)
	(1) pH				
	(2) hydrogen peroxide				
peptide	immobilizing peptides onto target proteins	cell membrane	nanofibers	promote the activation of T cells and improve T-cell-mediated tumor cytolysis	(63)

The design strategies employed for the supramolecular polymerization in biological systems generally center on controlling the patterning hydrophobic forces and π interactions at the structural level.^46^ As the affinity of these hydrophobic moieties is amplified in the aqueous environment in cells, they can serve as reactive nuclei that initiate assembly to circumvent their aversion to water. The directionality of their propagation into different morphologies depends on the extent to which the sequence and structure of each motif favors intermolecular alignment. In this respect, peptide amphiphiles, aromatic macrocycles, and their combinations are among the most robust candidates in which cooperative intermolecular forces, i.e., hydrogen bonds and electrostatic interactions, can be easily adjusted.^47−49^ In particular, assemblies based on short peptide sequences are already well known in biology due to the importance and abundance of α-helical (collagen, elastin) and β-sheet (amyloids) materials.^49^ Coupling the technology of exchanging sequences and synthetic groups in solid-phase peptide synthesis provides peptides with enormous diversity for structural control and design.^46^

**Figure 6:**
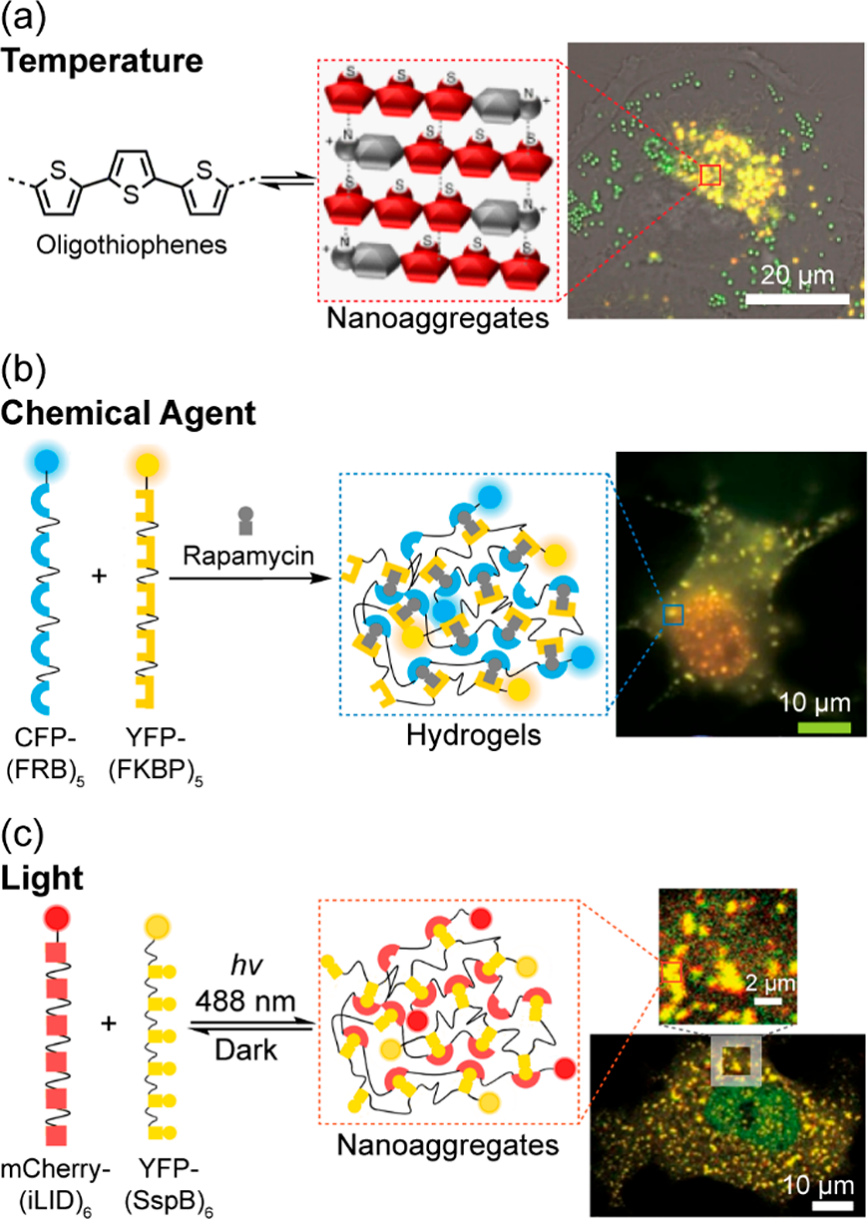
Extrinsically controlled assembly of aggregates in cells by different stimuli. (a) Temperature. Adapted with permission from ref (3). Copyright 2017 Springer Nature. (b) Chemical agent. (c) Light. (b and c) Adapted with permission from ref (53). Copyright 2017 Springer Nature.

While peptide-based scaffolds have so far represented the bulk of intracellular self-assembling materials, oligoaromatic compounds are making a unique entry into the field as they can provide a real-time fluorescence readout on their supramolecular behavior. This was first demonstrated with oligothiophenes that induce the formation of fluorescent fibrils within embryonic fibroblasts and HeLa cells.^50^ Deeper insights show that these oligothiophenes interact with pro-collagen peptides to form helices that made up the fibrillar architectures. Oligothiophene amphiphiles were separately developed by our group to enable organelle-specific compartmentalization.^3^ At the same time, spectral changes corresponding to the assembly state of the molecules were observed (Δλ_em_ ≈ 200 nm), which provided critical temporal information that correlated biological pathways with supramolecular behavior (Figure 6a). Unfortunately, the general accessibility of oligoaromatic compounds remains highly limited due to laborious synthetic routes with intimidating solubility problems, particularly in aqueous media.

Nevertheless, the recent development of supramolecular scaffolds aimed to achieve higher control over the assembly process, the subcellular location, initiation, and the time frame of polymerization, so that the biological impact of structure formation can be studied in more detail. In this sense, control can generally be achieved through two broad means: (1) by attaching a handle/caging group that temporarily disrupts the hydrophobic/hydrophilic balance until its cleavage or (2) by introducing a mechanism that switches structurally through isomerization, the inactive/active state of the assembly precursor molecule. Caging groups that are cleavable under specific conditions are well known in organic chemistry, with stimuli ranging from pH, temperature, redox, and light (Figure 6b and 6c),^51−53^ while enzyme-based triggers acting on peptides and sugars are correspondingly well established in molecular biology.^28,54−56^ In combination, these temporary groups can often be installed on supramolecular monomers to inhibit intermolecular forces and prevent premature assembly. Alternatively, structural switches are increasingly used with the assembly being dictated by the conformation of the assembling monomer. Exemplified by the Rao group, an inactive linear oligopeptide can be cyclized into its active form upon exposure to GSH and caspase 3/7.^57^ Separately, our group established the synthesis of ß-sheet-rich nanostructures in cells by an *N*-acyl rearrangement reaction that switches a peptide configuration from branched to linear and thus initiates nanostructure propagation.^2^ This rearrangement could also be coupled to a second reaction responding to reactive oxygen species (ROS), such that two chemical triggers, pH and ROS, must coexist for the reaction to occur. While these stimuli-responsive strategies provide an excellent platform to customize binding interactions with physiologically relevant cues, the dynamics of the living cell, i.e., intracellular transport and efflux, have yet to be overcome.

**Figure 7:**
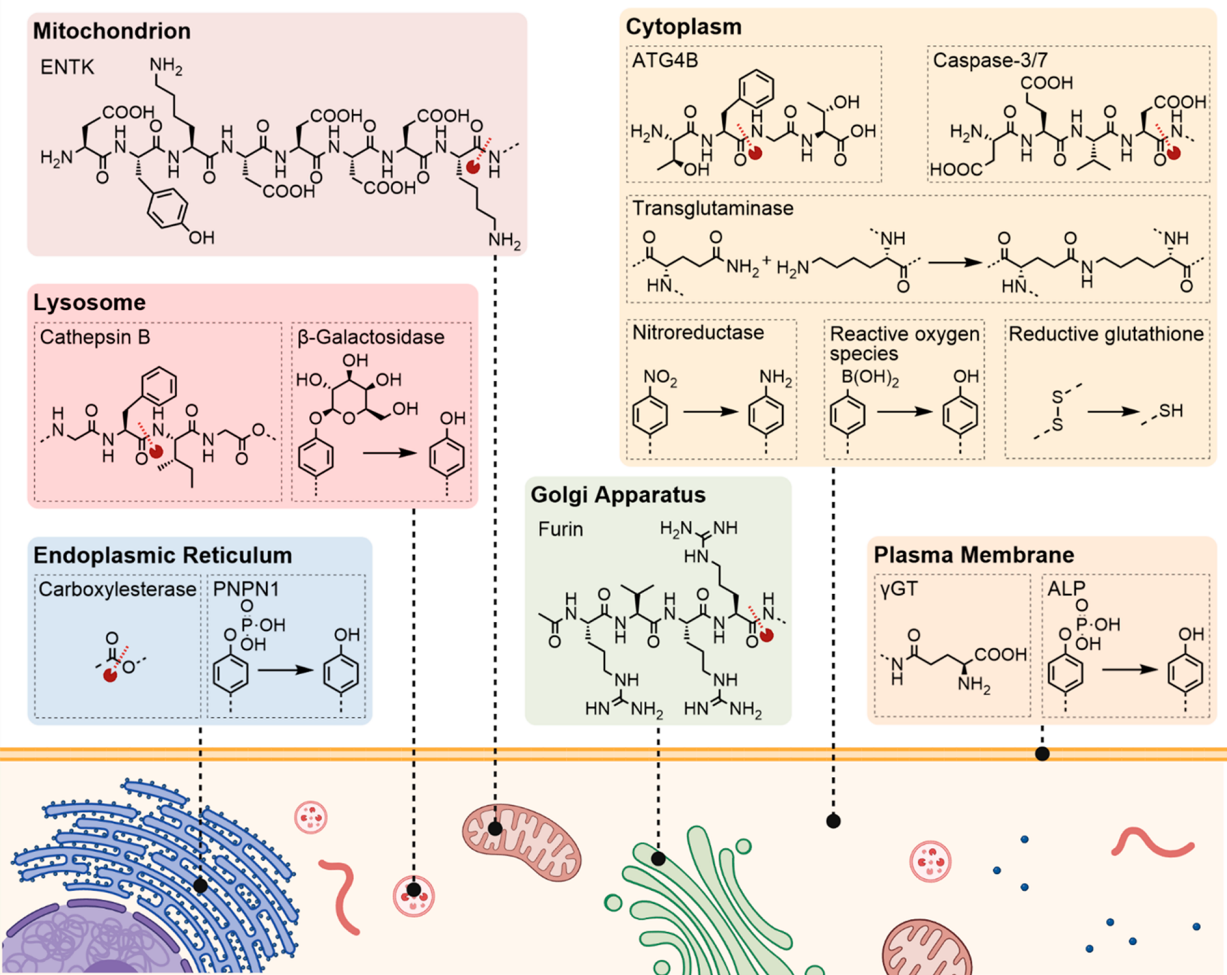
Selected intrinsic stimuli to induce intracellular assembly and their respective recognition and transformation sites.

In this respect, significant considerations include the cell targeting, toxicity, and stability of monomers that are crucial for successful supramolecular polymerization in cells. Targeting of monomers to a specific biological pathway or organelle can be achieved through the attachment of small molecular signaling handles, targeting peptides, or enzymatic recognition. Often, the conjugation of these motifs will also inherently suppress self-assembly as they interfere with the overall geometry and polarity of the molecule. Therefore, a streamlined design that is commonly found comprises a self-assembling unit connected to a targeting group via a cleavable linker (Figure 7). In this way, several criteria for intracellular assembly can be met simultaneously. Especially for peptidic scaffolds, motifs that respond to biological stimuli, such as the expression of enzymes including matrix metalloproteinases (MMPs), alkaline phosphatases (ALPs), esterases, and furin, were some of the first developed concepts.^16,17^ Initial studies relied on inserting phosphorylated tyrosine (pY) into amphiphilic peptide motifs and showed that the polarity shift upon dephosphorylation by ALP produces nanofibers in transformed *E. coli*.^54^ Further studies developed by different groups investigated tetrapeptide-cleavable substrates based on autophagy (-TFGF-) or enzymes overexpressed in cancer cells such as MMPs (-PLGL-) or caspase 3/7 (-DEVD-). In each of these studies, the general outcome is the stimulus-triggered release of an active self-assembling sequence that spontaneously forms a hydrogel-like material within the cell.^16,17^

Beyond enzymatic control, the concentration profile and distribution of monomers can also be leveraged to direct the formation of assemblies. Cellular transport mechanisms could be used to channel the accumulation of monomers in such a way that the critical assembly concentration is reached in the target compartment, in this case, the mitochondria.^58^ Using a pyrene-FFK tripeptide scaffold, the TPP moiety was attached to provide mitochondria localization. When the number of monomers exceeded the critical assembly threshold, fibers composed of amyloid cross-β-sheet structures formed spontaneously within the organelle. Other transport pathways such as via proteins (i.e., human serum albumin) could also be employed to facilitate cellular distribution and even reduce monomer toxicity, providing a useful tool for accessing a greater variety of assembly precursors.^3^

However, in comparison to enzyme-driven assemblies, other endogenous control mechanisms using ROS, GSH, pH, or concentration represent the minority. The lack of development is attributed to a synthetic chemical requirement to incorporate a switch to control the self-assembly behavior within the cell. Since the concentration of the stimuli can fluctuate depending on the cell types and subcellular compartments, it is challenging to pinpoint the exact chemical environment required to trigger the designed molecular switch.^14^ Among them, GSH-regulated redox cleavage has found the broadest inclusion since disulfide bonds can be integrated into peptide scaffolds with relative ease. Reactive oxygen species such as H_2_O_2_, which are also elevated in cancer cells, have recently been used to initiate assembly through the immolation of an aryl-boronic acid carbamate derivative. Subcellular localization based on pH gradients existing among cellular compartments such as the acidic endo/lysosomes, which are frequently used in drug delivery systems, has so far seen very limited application for intracellular assembly. Hence, there is ample room for developing intracellular self-assembly processes that are triggered by chemical cues, the completion of which would exhaust the full arsenal of tools available to study artificially driven dynamics in living systems.

## Impact of Supramolecular Architectures on Cellular Function

Following the initiation processes that trigger the formation of superstructures within cellular space, these assemblies provide a powerful tool for gaining a deeper understanding of transient architectures and phase-separated compartments in the living cells. Since the molecular structure of the precursor is defined, the extent of phase separation and its dynamics can be systematically studied by varying the substitution patterns on the monomer design. In this way, important insights could be obtained into how cellular architectures such as the cytoskeleton or RNA granules are assembled to perform their intended function.^59,60^ Photoactivated polymerization of a PEG-diacrylate monomer was reported for intracellular assembly and subsequent gelation in cells, which leads to immobilization of the cytosol but does not affect the fluidity of the cell membrane.^61^ The gelation of antigen-presenting immune cells was investigated as a possible application, where they could interact with T cells comparable to living cells, which subsequently led to the expansion of the T cells. Besides full gelation of the cytoplasm, partial gelation can be used to mimic liquid–liquid phase separation within cells. Such phase separation behavior can also be exhibited by protein–peptide conjugates that are expressed by cells and form hydrogels by chemically induced dimerization. The interaction of two proteins was induced by the addition of an effector molecule or by light irradiation, resulting in chain linking of the protein–peptide constructs and subsequent formation of hydrogels (Figure 6b and 6c).^53^ A different approach to mimic liquid–liquid phase separation to achieve simultaneous sequestration of two enzymes was developed using peptide precursors functionalized with protein ligands.^62^ Upon an enzyme-instructed self-assembly (EISA) of the peptide, the obtained superstructure resulted in a high local concentration of two proteins.

Besides the fundamental importance of learning how Nature utilizes superstructures, the concept has also fueled the development of revolutionary strategies that could potentially address long-standing limitations of conventional therapeutics. Accumulation of RGD-modified peptide on tumor cells with overexpressed α_v_β_3_ receptor led to the formation of nanofibers that promote the activation of T cells and improve T-cell-mediated tumor cytolysis.^63^ Expression of cancer-associated enzymes such as MMP-7 or ALP can be utilized for enzymatic activation of peptide precursors with high spatiotemporal control to form superstructures for cancer therapy.^16,17^ A peptide precursor containing an MMP-7 cleavage site was reported, which showed high specificity toward cancer cells when cocultured with healthy cells, and explained that the stress induced by the superstructures leads to cell death.^43^ Other studies demonstrated that self-assembly at the mitochondria in cancer cells resulted in their dysfunction and apoptosis.^58^ Recently, EISA and organelle targeting were combined in a tetrapeptide self-assembled near the mitochondria, which induces the disruption of mitochondria membranes by the superstructures and subsequent release of cytochrome *c* to induce apoptosis.^64^ The incorporation of subcellular targeting helps to understand the functionality of different organelles inside cells and paves the way toward new cancer therapeutics based on supramolecular assemblies with a lower risk for developing resistance to conventional chemotherapeutics.^65^

Separately, our group demonstrated the rational design of an isopeptide precursor that chemically transforms into a linear self-assembling sequence upon elevated reactive oxygen species within cancer cells.^2^ Cellular uptake of the pro-assembling peptide was mediated by a cell-penetrating peptide where the first stage was controlled by a pH-sensitive dynamic covalent bond using boronic acid/salicyl hydroxamate chemistry. Its dissociation in the acidic endosomes subsequently revealed the second stage, which undergoes immolation in the presence of endogenous hydrogen peroxide in A549 lung adenocarcinoma cells. Once the functionalities that were inhibiting the assembly were removed, the kinked peptide spontaneously rearranged via an *O,N*-acyl shift that linearized the backbone, thus propagating nanofiber assembly. The formation of the nanofibers was observed to induce downstream pathways such as the disruption of cytoskeleton integrity and apoptosis.

Our group further introduced a near-infrared photoluminescence-emitting Pt(II)–terpyridine complex to the isopeptide platform.^1^ Through the cascade transformation induced by acidic endosomal pH and cytoplasmic hydrogen peroxide, nanofibers were formed intracellularly in which the Pt(II) complex directs the supramolecular order and directionality of the packing within the fiber axis. Formation of the nanofibers damages energy homeostasis and essential metabolic pathways within the cancer cells, thus preventing the cells from mounting adaptive strategies that are known to resist specific small-molecule inhibitors and increase metastasis. The mechanistic origin of the disruption is confirmed by impaired ATP-dependent pathways, including actin growth and histone deacetylase activities. The intracellular formation of nanofibers was found to induce apoptosis and leverage a similar potency on various cell types. This study demonstrated that the directed assembly of nanostructures within cancer cells could be employed as a general strategy for designing metabolically active materials to induce systemic level effects and compensate for the limitations of small molecules and biologics.

From a chemical perspective, synthetic functional groups integrated into biologically triggered systems can provide a complementary breadth of information toward an observed physiological response. Inspired by the synthesis of d-luciferin, monomers that perform an intramolecular reaction between 1,2-aminothiols and 2-cyanobenzothiazole (CBT) were developed.^57^ The obtained macrocyclic compound formed fluorescent nanostructures due to hydrophobic interactions and π–π stacking. Peptide caging groups were linked to the amino functionality, allowing a correlation between fluorescence and enzymatic activity. Optimizations of the system were investigated by altering caging groups as well as tuning the spacer between the reactive 1,2-aminothiols and CBT.^47,66^

## Conclusions and Outlook

In summary, we have presented in this Account an insight toward forming synthetic nanoscale structures in living cells, its complexity within the dynamics of intracellular transport, and its immense potential to modulate biological responses. Depending on whether covalent or noncovalent bonds are formed, different chemical perspectives are explored together with how a designated reaction can be reliably performed in a variety of pathways and cellular components. The overarching concept relies upon a thorough understanding of cell biology together with state-of-the-art techniques that often test the limits of synthetic chemistry. While significant headway has demonstrated that polymerizations and self-assembly processes in living systems are no longer exclusive to Nature, current methods still lack the precision to establish accurate structure–activity or sequence–activity relationships. In most cases, the polymerization reaction or assembly in cells cannot be stopped at a defined length or size and will run its due course.

Similarly, modern characterization techniques would also require further improvement to visualize these processes in higher definition, in real time, and in the native state of the cell. The main challenge is that the considerations from the synthetic and characterization sides are largely intertwined, such as when a fluorescent label is required for visualization but its presence would alter the polymerization process. As such, a collective and communal effort is required to circumvent these limitations by the innovation of synthetic and technical methodologies. By building on these capabilities, we will be equipped to explore and unravel the next level of intricacies in the chemistry of life.
